# Complete Genome Sequence of the *Mycobacterium immunogenum* Type Strain CCUG 47286

**DOI:** 10.1128/genomeA.00401-16

**Published:** 2016-05-26

**Authors:** Daniel Jaén-Luchoro, Carolina Seguí, Francisco Aliaga-Lozano, Francisco Salvà-Serra, Antonio Busquets, Margarita Gomila, Antonio Ramírez, Mikel Ruiz, Edward R. B. Moore, Jorge Lalucat, Antoni Bennasar-Figueras

**Affiliations:** a Microbiologia, Departament de Biologia, Universitat de les Illes Balears, Palma de Mallorca, Illes Balears, Spain; b Microbiologia, Clínica Rotger, Palma de Mallorca, Illes Balears, Spain; c Culture Collection University of Gothenburg (CCUG), Sahlgrenska Academy, University of Gothenburg, Gothenburg, Sweden; d Department of Infectious Diseases, Sahlgrenska Academy, University of Gothenburg, Gothenburg, Sweden; e Servicio de Microbiología, Hospital Universitari Son Espases, Palma de Mallorca, Illes Balears, Spain

## Abstract

Here, we report the complete genome sequence of *Mycobacterium immunogenum* type strain CCUG 47286, a nontuberculous mycobacterium. The whole genome has 5,573,781 bp and covers as many as 5,484 predicted genes. This genome contributes to the task of closing the still-existing gap of genomes of rapidly growing mycobacterial type strains.

## GENOME ANNOUNCEMENT

Nosocomial infections with nontuberculous mycobacteria (NTM) have acquired an important role due to the inherent clinical problems caused by several of its members. Among these bacteria, *Mycobacterium immunogenum* is a nonpigmented and rapidly growing mycobacterium (RGM). Strain CCUG 47286^T^ (= CIP 106684^T^ = MC 779^T^), isolated from waterborne contamination of a bronchoscope washer in St. Louis, MO, USA, was described as a new species closely related to *Mycobacterium abscessus* and *Mycobacterium chelonae* (1).

Several strains identified as *M. immunogenum* stand out for their ubiquity and implications in nosocomial infections (2), including pseudo-outbreaks in bronchoalveolar lavage procedures (3), skin infections (4), mesotherapy treatment infections (5), and hypersensitivity pneumonitis (6). In 2006, the first keratitis outbreak associated with *M. immunogenum* was reported (3). Although NTM are not commonly associated with disseminated infections, in 2012, the first reported case was published of a patient with septic shock derived from a disseminated infection of *M. immunogenum* (2). Additionally, its high resistance to antibiotics (1) and capability to grow and disseminate through water system suppliers (7) make *M. immunogenum* an important opportunistic pathogen of the NTM group.

Bulk genomic DNA (gDNA) was prepared from *M. immunogenum* CCUG 47286^T^ using a protocol optimized for mycobacteria, combining the Wizard genomic DNA purification kit (Promega, Spain) and mechanical disruption with Disruptor Genie (Scientific Industries, Inc., USA). The gDNA was used for paired-end (PE) library preparation with the Illumina genomic Nextera XT kit, followed by sequencing on a HiSeq 2500 platform. The genome was also sequenced with a Pacific Biosciences (PacBio) RSII single-molecule real-time (SMRT) platform. PacBio 10-kb SMRTbell libraries were prepared, according to the protocols of the manufacturer.

A hybrid Illumina-PacBio *de novo* assembly protocol was applied to obtain a highly continuous genome. Briefly, high-quality Illumina reads were obtained by filtering and enhancement with BBMap version 35.34 (http://sourceforge.net/projects/bbmap). The ABySS software, version 1.5.1 (8), was used for an initial *de novo* assembly. The initial contigs and 61,476 PacBio reads (average read length, 2,677 bp), after being processed and filtered using the SMRT Analysis pipeline version 2.2, were aligned with BLASR (9). This alignment was used to estimate the orientation, arrangement, and distances between contigs (10). The gap regions were partially closed by iteratively passing 2,863,242 PE (100-bp) Illumina reads (11). A BLAST database was generated from contigs previously assembled using Velvet version 1.1.04 (12) and high-quality 100-bp PE Illumina reads, at 50× coverage. This searchable database was used to confirm and close the remaining gaps and resolve conflicting assembled regions. The fully closed genome had a final coverage of 125×.

The *M. immunogenum* genome has 5,573,781 bp and 64.3% G+C content. Two complete ribosomal operons and 61 tRNAs were detected after annotation with the Prokaryotic Genome Annotation Pipeline (PGAP) of the NCBI (13). After RAST analysis (14), from the predicted 5,484 coding sequences (CDSs), 1,825 (34%) were included in 407 subsystems. Among them, 109 CDSs were related to subsystems associated with virulence, disease, and defense; 33 were related to resistance to antibiotics and toxic compounds; and 79 were related to invasion and intracellular resistance processes. Additionally, two potential toxin-antitoxin systems were detected.

### Nucleotide sequence accession number.

The complete genome sequence of *M. immunogenum* type strain CCUG 47286 has been deposited in DDBJ/ENA/GenBank under the accession no. CP011530. The version described in this paper is the first version.
